# Understanding Recession and Self-Rated Health with the Partial Proportional Odds Model: An Analysis of 26 Countries

**DOI:** 10.1371/journal.pone.0140724

**Published:** 2015-10-29

**Authors:** Adam Mayer, Michelle Foster

**Affiliations:** 1 Department of Sociology, Colorado State University, Fort Collins, Colorado, United States of America; 2 Department of Food Science and Human Nutrition, Colorado State University, Fort Collins, Colorado, United States of America; Universidad Veracruzana, MEXICO

## Abstract

**Introduction:**

Self-rated health is demonstrated to vary substantially by both personal socio-economic status and national economic conditions. However, studies investigating the combined influence of individual and country level economic indicators across several countries in the context of recent global recession are limited. This paper furthers our knowledge of the effect of recession on health at both the individual and national level.

**Methods:**

Using the Life in Transition II study, which provides data from 19,759 individuals across 26 European nations, we examine the relationship between self-rated health, personal economic experiences, and macro-economic change. Data analyses include, but are not limited to, the partial proportional odds model which permits the effect of predictors to vary across different levels of our dependent variable.

**Results:**

Household experiences with recession, especially a loss of staple good consumption, are associated with lower self-rated health. Most individual-level experiences with recession, such as a job loss, have relatively small negative effects on perceived health; the effect of individual or household economic hardship is strongest in high income nations. Our findings also suggest that macroeconomic growth improves self-rated health in low-income nations but has no effect in high-income nations. Individuals with the greatest probability of “good” self-rated health reside in wealthy countries ($23,910 to $50, 870 GNI per capita).

**Conclusion:**

Both individual and national economic variables are predictive of self-rated health. Personal and household experiences are most consequential for self-rated health in high income nations, while macroeconomic growth is most consequential in low-income nations.

## Background

Socio-economic conditions- such as personal income or national economic development- are demonstrated to be determinants of health [1–6] as access to resources that individuals can use to improve their health status is influenced by economic conditions [7,8]. However, the impact of the recessions, such as the financial crisis in the U.S. housing market in 2007 that quickly accelerated internationally, on health outcomes remains poorly understood due, to some degree, to variability in the effect of recession by unit of analysis [9–12] as explained below.

Research demonstrates that recessions are associated with *improved* health at the level of an aggregate or ecological unit (e.g. country or other large unit). For instance, in a sample of U.S. counties self-rated health is positively associated with unemployment [11]. In opposition, mortality increases at the national level during periods of economic expansion [6,9]. Although it is unclear why recessions are positively correlated with improved health at an aggregate level, it is proposed that recessions are associated with reductions in unhealthy behaviors such as smoking and drinking [12–14] and stress-related cardiovascular illness [15]. That is, people might reduce their consumption of alcohol and tobacco and have less work induced stress during economic hard times, thereby leading to aggregate improvements in mortality.

The effect of economic recession at the individual level has been demonstrated to move in the opposite direction as those of an aggregate unit (e.g. a nation), such that economic crisis, or associated consequences like unemployment, reduces health when measured at the level of individuals. For example, in Greece [10] and Sweden [16] self-rated health declined during the 2007 recession and mental health deteriorated in the UK during and after the recession [17]. Causes for this association remain unclear, but research proposes that, at the individual level, recessions create perceptions of economic insecurity, which in turn reduces self-rated health [18–23].

Evidence also suggests that the effect of macroeconomic change on perceived health varies across countries. Countries with high levels of economic development and strong social safety nets do not have severe reductions in perceived health following recessions [24]. Sharp cuts to public services and social welfare expenditures may exacerbate the effects of recession on health [25], which may explain why the recent global recession had much more deleterious health consequences for some countries than others. Conversely, the effect of macroeconomic growth on self-rated health is proposed to vary by the level of economic development of a country [9]. More specifically, economic growth is suggested to improve health in lower income nations, but have little effect or a negative effect in high-income nations.

Taken together, it appears that direction of the relationship between recession and health varies upon the unit of analysis (e.g. aggregate vs. individual). Hence, researchers should consider both aggregate level predictors, such as country level economic variables, and individual level variables, such as measures of job loss, to systematically scrutinize the health consequences of recession. This article examines the effect of macro-economic change and personal experiences with the recent global recession on self-rated health by using a sample of 26 European countries. In addition, we employ a unique modeling strategy (partial proportional odds models) which allow for more nuanced insights about the effect of economic change than more popular approaches, such as the ordinal logistic model. Specifically, the partial proportional odds model allows the effect of predictor variables to vary across categories of the dependent variable.

## Methods

### Data and Variables

Data from the Life in Transition II (LITS II) international survey [26] was used to examine the relationship between self-rated health and recession. LITS II, conducted in 2010, was a joint project between the European Central Bank and World Bank. LITS II included questions pertaining, but not limited, to experiences with the global recession of 2007–2008. LITS II data analysis comprised a multi-stage random probability stratified cluster sampling design. A standardized instrument was translated into the appropriate languages for each country and pre-testing was conducted in each country. LITS II screened respondents within households using a combination of the Kish and nearest-birthday method. Response rates varied across countries (e.g. 8% in Slovakia to 70% in Albania), but when weighted by country-specific sample size the overall response rate was 37.7%. Our sample includes the following 26 countries: Albania, Belarus, Bosnia and Herzegovina, Bulgaria, Croatia, Czech Republic, Estonia, France, Germany, Great Britain, Hungary, Italy, Kosovo, Latvia, Lithuania, Macedonia, Moldova, Montenegro, Poland, Romania, Russia, Serbia, Slovakia, Slovenia, Sweden and Ukraine.

Following prior research [27–29] self-rated health is the outcome variable. Respondents were asked: “How would you assess your health?” using a 1–5 scale (Very Bad, Bad, Medium, Good and Very Good). These variables were recoded into 3 categories; these where 1 = poor, 2 = moderate and 3 = good self-rated health due to data sparseness caused by low response in certain variable categories. Respondents who answered “very bad” or “bad” in the original variable were collapsed into the “poor” category. Respondents who answered “very good” or “good” were collapsed into the “good” category. The “medium” category of the original variable was not recoded. Evidence indicates that objective health status is strongly associated with self-rated health. For example, individuals report improved self-rated health after a change in objective health status, such as voluntary weight loss [29]. Obesity and mortality are negatively correlated with self-rated health [30–33]. Thus, despite the subjective nature of self-rated health it is a valid indicator of actual health status [34].

This analysis includes a measure of the percentage change in gross national income from 2009–2010, provided by the World Bank database [35], at the country level. At the individual level we include a number of variables to capture experiences with the recession and a series of control variables (all individual level variables come from the Life in Transition II study). The recession experience variables include: the head of household experienced a job loss, other members of the household experienced a job loss, the respondent experienced a wage cut, and the respondent or anyone in their household had to reduce their consumption of either luxury or staple goods. Each is coded so that “1” represents experiencing the event. We also employ a series of socio-demographic control variables, which include a seven-category measure of education (1 = no education to 7 = PhD), a binary variable for sex (1 = male, 2 = female) and a six-category indicator for a respondent’s age (1 = 18–24 to 6 = 65+). Our data does not include a variable for household income that is comparable across countries. Instead, we use a measure of subjective income, in which respondents were asked to rank the income decile that their household belongs in. This variable was recoded because of low response in high or low-income decile categories. Respondents who answered 1–3 on this variable were recoded into 1 (low), those who stated 4–6 were recoded into category 2 (middle) and those who answered 7–10 were recoded into category 3 (high). Thus, the recoded categories are low(1), middle(2) and high (3) income. It is likely that access to medical care influences self-rated health so a binary variable was included to capture utilization of health care. More specifically, respondents were asked if anyone in their household had received medical care in the past year. In this variable 0 = no and 1 = yes. More information about the variables can be found in Table 1.

**Table 1:** Question Wordings and Descriptions for Self-Rated Health and Recession Experience Variables.

Variable name	Question wording or description	Source
Self-rated Health	How would you assess your health?	Life in Transition II
GNI growth(%)	Percent growth in GNI per capita from 2009–2010	World Bank
Job loss-head of household	How has this economic crisis affected you (or other household members) in the past two years? Head of household lost job	Life in Transition II
Job loss-other member of household	How has this economic crisis affected you (or other household members) in the past two years? Other member of household lost job	Life in Transition II
Wage Reduction	How has this economic crisis affected you (or other household members) in the past two years? Wages reduced	Life in Transition II
Staple Consumption Reduction	In the past two years, have you or anyone else in your household had to take any of the following measures as the result of a decline in income or other economic difficulty?, Reduced consumption of staple foods such as milk / fruits / vegetables / bread	Life in Transition II
Luxury Consumption Reduction	In the past two years, have you or anyone else in your household had to take any of the following measures as the result of a decline in income or other economic difficulty? Reduced consumption of luxury goods	Life in Transition II
Age	Respondent's Age	Life in Transition II
Education	What is the highest level of education you have already completed?	Life in Transition II
Social Class	Recoded from: Please imagine a ten-step ladder where on the bottom, the first step, stand the poorest 10% people in our country, and on the highest step, the tenth, stand the richest 10% of people in our country. On which step of the ten is your household today?	Life in Transition II
Healthcare Use	In the past 12 months has this household used healthcare services?	Life in Transition II
Female	Respondent's sex	Life in Transition II

### Modelling Strategy

Before we estimated the statistical models we conducted a descriptive analysis reported in the next section. After this descriptive analysis, a regression-based approach was implemented to examine the effects of person-level and national-level economic experiences on self-rated health. For ordinal outcomes (such as self-rated health) it is traditional to use an ordinal logistic or ordinal probit modeling strategy, but these models assume that coefficient do not vary across cut point equations [36–37]. That is, the ordinal model assumes that effects of independent variables are equal at different thresholds (i.e. categories) of the dependent variable [38]. This “proportional odds” assumption is often violated, which indicates that ordinal logistic or ordinal probit regression models are not appropriate. Consequently, to address this problem partial proportional odds models, also referred to as a generalized ordinal logistic models [39–42], was executed in Stata 13/ SE [43] using the *gologit2* package developed by Richard Williams [41]. The partial proportional odds model is advantageous relative to other possible modelling strategies, such as the multinomial logistic model, because not all variables are likely to violate the proportional odds assumption. With this model the proportional odds assumption is relaxed for those variables that violate the assumption, providing a much more parsimonious model than a multinomial logistic model and a more accurate model than an ordinal model. In the first part of the model, coefficients represent the effect of a one-unit change in the respective independent variable on the log-odds of a particular category compared to all other categories of the dependent variable. Then, the partial proportional odds model estimates additional coefficients for predictors which have violated the proportional odds assumption. Williams [41] explains the coefficients in this manner: “Hence, positive coefficients indicate that higher values on the explanatory variable make it more likely that the respondent will be in a higher category of Y than the current one, whereas negative coefficients indicate that higher values on the explanatory variable increase the likelihood of being in the current or a lower category.” (p. 63). Williams [41] provides the following representation of this model:

$$P(Y_{i}>j)=\frac{\text{exp}(a_{j}+{X1}_{i},B1+X2,B2+X3,{B3}_{j})}{1+\text{exp}(a_{j}+{X1}_{i},B1+X2,B2+X3,{B3}_{j})}j=1,2\ldots .M-1$$

Hence, the probability of a particular category of the dependent variable (*Y*_i)_ is a function of coefficients represented with betas which may freely vary across values of *j-* which denotes the number of categories of the outcome variable. Here, *M* signifies the number of categories of the outcome variable. Thus, it is possible that the partial proportion odds model may produce up to *M*-1 coefficients for each predictor variable. Typically, a Wald statistic test is used to determine if a predictor violates the proportion odds assumption; in this paper we use the conventional cut-off of p = .05.

We estimate five unique partial proportional odds models. The first model pools data for all 26 countries and the next series of models are disaggregated into quartiles of 2010 GNI per capita. GNI per capita in the first quartile ranges from $1820-$4640 and includes the following countries: Albania, Bosnia-Herzegovina, Bulgaria, Macedonia, Moldova, Ukraine and Kosovo. The second GNI per capita quartile ranges from $5550-$10,100 and includes the following nations: Belarus, Croatia, Montenegro, Romania, Russia, and Serbia. The third quartile has a GNI per capita range of $12,330-$18,450 and includes these countries: Czech Republic, Estonia, Latvia, Lithuania, Poland, and Slovakia. Finally, the fourth GNI per capita quartile ranges from $23,910 to $50,870 and includes the following nations: France, Germany, Great Britain, Italy, Slovenia, and Sweden. We provide predicted probabilities after we present the modelling results.

## Results

### Descriptive Analysis

Table 2 provides the percentage of respondents reporting each category of self-rated health by country. Sweden (77%) and Montenegro (75%) and lead the “good” category. Russia (53%), Belarus and Ukraine (51%) lead the middle category. Low self-rated health is most common in Romania (26%) and Lithuania (21%). Overall, a small majority (55%) of respondents reported “good” self-rated health.

**Table 2:** Self Rated Health by Country.

	Poor	Moderate	Good
	%	%	%
Albania	6	21	73
Belarus	10	51	39
Bosnia	14	22	64
Bulgaria	15	35	50
Croatia	15	31	55
Czech Republic	9	31	60
Estonia	17	45	38
France	5	23	72
Germany	6	24	69
Great Britain	10	23	67
Hungary	19	39	42
Italy	4	27	69
Kosovo	5	16	80
Latvia	17	46	37
Lithuania	21	44	36
Macedonia	9	28	63
Moldova	31	36	33
Montenegro	8	17	75
Poland	15	27	58
Romania	26	31	43
Russia	13	53	33
Serbia	17	28	55
Slovakia	6	26	68
Slovenia	9	27	64
Sweden	7	16	77
Ukraine	17	51	32
All Countries	13	32	55

The percentage of respondents answering “yes” to the recession experience variables and the percentage change in GNI per capita in each country is reported in Table 3. The most striking finding of Table 3 is that respondents reported negative experiences with the recession in countries that were not formally in recession when the survey was administered. For example, GNI increased by 15% in Moldova but 30% of respondents indicated that the head of the household had lost their job. Hence, household experiences with the global recession may be only loosely related to whether the nation actually experienced a deep recession by official definitions (i.e. two consecutive quarters of no economic growth). The nations with both strong economic growth and a great deal of respondents reporting negative experiences with the recession have low GNI per capita. We speculate that these countries are likely to have relatively weak social safety nets that cannot effectively redistribute the potential benefits of economic growth to the population at large [44]. Table 4 provides the percentages of respondents in each category for the control variables. Percentage distribution of control variables for individual countries is shown in S1 Appendix.

**Table 3:** Recession Experience by Country.

	Jobloss-head of household	Jobloss-other member of household	Wage Reduction	Staple Consumption Reduction	Luxury Consumption Reduction	GNI Change
	%yes	%yes	%yes	%yes	%yes	%
Albania	19	24	22	36	58	0.25
Belarus	5	6	65	26	33	7.16
Bosnia	10	13	40	23	47	-0.42
Bulgaria	13	16	31	56	61	3.95
Croatia	13	17	67	36	54	-0.15
Czech Republic	8	12	43	22	30	2.62
Estonia	14	15	42	26	43	-0.77
France	7	9	27	13	32	-0.45
Germany	9	11	18	8	26	1.74
Great Britain	11	7	20	10	32	-6.82
Hungary	11	11	22	57	49	-0.92
Italy	5	12	36	19	57	-0.06
Kosovo	19	23	48	33	65	3.08
Latvia	26	22	69	56	42	-4.36
Lithuania	14	11	57	41	53	-0.68
Macedonia	21	32	39	52	67	2.86
Moldova	30	27	46	48	53	15.92
Montenegro	10	15	61	31	57	2.37
Poland	6	12	33	21	27	2.13
Romania	11	24	62	45	47	-3.64
Russia	14	15	63	35	30	6.57
Serbia	12	16	49	46	61	-1.75
Slovakia	13	16	34	13	27	1.33
Slovenia	8	11	58	21	62	0.67
Sweden	6	5	16	4	19	3.99
Ukraine	14	18	53	53	42	5.28
All Countries	13	16	44	32	45	1.50

**Table 4:** Percent distribution of control variables.

Age	%
18–24	10.26
25–34	18.1
35–44	17.85
45–54	17.5
55–64	16.69
65+	19.59
Education	
No education	6.11
Primary	3.64
Lower Secondary	11.4
Upper Secondary	18.67
Post Secondary, Non-Tertiary	29.67
Bachelor's degree or more	16.32
Master's degree or PhD	14.2
Social Class	
Low	29.36
Middle	66.64
High	4
Access	
No	32.95
Yes	67.05
Female	
Male	40.45
Female	59.55

### Modelling results

Log-odds coefficients, standard errors, and p-values for the models are presented in Tables 5–9. We note which variables have violated the proportional odds assumption in the footnotes of each table. For simplicity we only report two coefficients for variables that were found to violate the assumption instead of presenting two coefficients for all variables. For example, the variable female in Table 5 was found to be in violation of the proportion odds assumption via Wald tests, thus we provide a coefficient in the column which compares “good” vs. “moderate and poor” and another which compares “good and moderate” versus “poor” health. No coefficient in the “good and moderate” versus “poor” health column means that the effect of that particular variable is symmetrical across categories of the self-rated health. That is, the effect is the same for “good” vs. “moderate” and “poor” and “good and moderate” vs. “poor”. The p-values associated with the Wald tests for each model can be found in S2 Appendix; a significant test indicates that the proportional odds assumption has been violated. In S3 Appendix we present multicollinearity diagnostics. These diagnostics indicate that multicollinearity is not a problem in our models. In addition, each model employs appropriate sampling weights to adjust for non-response and missing data is excluded from the analysis.

**Table 5:** Weighted Partial Proportional Odds Model of Self-Rated Health, all countries.

	Good vs. moderate or poor			Good and moderate vs. poor		
	coef	std. error	p	coef	std. error	p
**Country Level Variables**						
GNI growth(%)	0.057	0.004	0.000			
**Recession Experience Variables**						
Jobloss-head of household	0.226	0.046	0.000			
Jobloss-other member of household	0.140	0.042	0.001			
Wage Reduction	0.215	0.034	0.000	-0.071	0.049	0.151
Staple Consumption Reduction	0.438	0.032	0.000			
Luxury Consumption Reduction	-0.137	0.031	0.000			
**Controls and Socio-Demographics**						
Age						
25–34	0.244	0.072	0.001			
35–44	0.739	0.069	0.000			
45–54	1.491	0.067	0.000			
55–64	2.083	0.068	0.000			
65+	2.620	0.070	0.000			
Education						
Primary	0.259	0.103	0.012	-0.133	0.107	0.215
Lower Secondary	0.003	0.099	0.979	-0.460	0.107	0.000
Upper Secondary	-0.322	0.096	0.001	-0.823	0.107	0.000
Post Secondary, Non-Tertiary	0.003	0.100	0.975	-0.795	0.114	0.000
Bachelor's degree or more	-0.413	0.102	0.000	-1.159	0.127	0.000
Master's degree or PhD	-0.393	0.115	0.001	-1.072	0.161	0.000
Social Class						
Middle	-0.559	0.033	0.000	-0.609	0.180	0.001
High	-1.126	0.110	0.000			
Access	0.556	0.034	0.000	0.556	0.034	0.000
Female	0.395	0.034	0.000	0.287	0.050	0.000
Constant		-1.858			-3.373	
Psuedo R^2^	0.165					

n = 19759, Countries: Albania, Belarus, Bosnia and Herzegovina, Bulgaria, Croatia, Czech Republic, Estonia, France, Germany, Great Britain, Hungary, Italy, Kosovo, Latvia, Lithuania, Macedonia, Moldova, Montenegro Poland, Romania, Russia, Serbia, Slovakia, Slovenia, Sweden and Ukraine. The proportional odds assumption was violated for GNI growth (%), Wage Reduction, Education (all categories), and Social Class (middle).

**Table 6:** Weighted Partial Proportion Odds Model of Self-Rated Health, First GNI per capita quartile ($1820-$4640).

	Good vs. moderate or poor			Good and moderate vs. poor		
	coef.	std. error	p	coef.	std. error	p
**Country Level Variables**						
GNI growth(%)	0.100	0.008	0.000	0.079	0.009	0.000
**Recession Experience Variables**						
Jobloss-head of household	0.062	0.080	0.436			
Jobloss-other member of household	0.063	0.073	0.394			
Wage Reduction	0.043	0.063	0.496			
Staple Consumption Reduction	0.290	0.062	0.000			
Luxury Consumption Reduction	-0.144	0.062	0.020			
**Controls and Socio-Demographics**						
Age						
25–34	0.138	0.131	0.291			
35–44	0.742	0.127	0.000			
45–54	1.359	0.153	0.000	1.713	0.153	0.000
55–64	2.099	0.129	0.000			
65+	2.705	0.135	0.000			
Education						
Primary	-0.455	0.154	0.003			
Lower Secondary	-0.516	0.153	0.001			
Upper Secondary	-0.660	0.152	0.000	-0.917	0.172	0.000
Post Secondary, Non-Tertiary	-0.365	0.154	0.018	-1.104	0.173	0.000
Bachelor's degree or more	-0.587	0.161	0.000	-1.518	0.207	0.000
Master's degree or PhD	-1.114	0.269	0.000	-2.620	0.734	0.000
Access	0.528	0.073	0.000			
Female	0.515	0.068	0.000	0.284	0.097	0.004
Social Class						
Middle	-0.679	0.064	0.000			
High	-1.411	0.219	0.000			
Constant		-1.653			-3.404	
Psuedo R^2^	0.201					

n = 5318, Countries: Albania, Bosnia-Herzogovina, Bulgaria, Macedonia, Moldova, Ukraine and Kosovo. The proportional odds assumption was violated for Age (45–54), Education (Upper Secondary, Post Secondary, Bachelor's degree, Master's degree, Female)

**Table 7:** Partial Proportional Odds Model for Self-Rated Health, second GNI per capita quartile ($5550-$10,100).

	Good vs. moderate or poor			Good and moderate vs. poor		
	coef.	std. error	p	coef.	std. error	p
**Country Level Variables**						
GNI growth(%)	0.113	0.010	0.000	-0.025	0.014	0.076
**Recession Experience Variables**						
Jobloss-head of household	0.195	0.099	0.050			
Jobloss-other member of household	0.170	0.086	0.048			
Wage Reduction	0.007	0.066	0.910			
Staple Consumption Reduction	0.377	0.066	0.000			
Luxury Consumption Reduction	-0.153	0.064	0.017			
**Controls and Socio-Demographics**						
Age						
25–34	0.377	0.140	0.007			
35–44	0.916	0.138	0.000	0.527	0.210	0.012
45–54	1.740	0.139	0.000	1.385	0.179	0.000
55–64	2.366	2.366	0.000	2.058	0.168	0.000
65+	2.621	0.155	0.000			
Education						
Primary	-0.238	0.194	0.220			
Lower Secondary	-0.535	0.196	0.007			
Upper Secondary	-1.000	0.187	0.000			
Post Secondary, Non-Tertiary	-0.508	0.203	0.012	-0.929	0.230	0.000
Bachelor's degree or more	-1.009	0.200	0.000			
Master's degree or PhD	-0.506	0.236	0.032			
Social Class						
Middle	-0.453	0.074	0.000	-0.686	0.097	0.000
High	-0.931	0.232	0.000			
Access	0.469	0.072	0.000			
Female	0.534	0.069	0.000	0.281	0.099	0.004
Constant		-1.297			-2.919	
Psuedo R^2^		0.188				

n = 4669, Countries: Belarus, Croatia, Montenegro, Romania, Russia, and Serbia. The proportional odds assumption was violated for GNIchange, Age (35–44, 45–54, 55–64) Education (Post-Secondary), Social Class (Middle) and Female.

**Table 8:** Weighed Partial Proportion Odds Model of Self-Rated Health, third GNI per capita quartile ($12,330-$18,450).

	Good vs. moderate or poor			Good and moderate vs. poor		
	coef.	std. error	p	coef.	std. error	p
**Country Level Variables**						
GNI growth(%)	-0.116	0.017	0.000	0.020	0.022	0.351
**Recession Experience Variables**						
Jobloss-head of household	0.231	0.086	0.007			
Jobloss-other member of household	0.114	0.082	0.163			
Wage Reduction	0.077	0.069	0.266	-0.303	0.093	0.001
Staple Consumption Reduction	0.276	0.070	0.000	0.470	0.087	0.000
Luxury Consumption Reduction	-0.104	0.058	0.075			
**Controls and Socio-Demographics**						
Age						
25–34	0.551	0.143	0.000			
35–44	1.129	0.137	0.000			
45–54	2.028	0.135	0.000			
55–64	2.771	0.142	0.000	2.460	0.163	0.000
65+	3.518	0.152	0.000	3.089	0.157	0.000
Education						
Primary	0.519	0.392	0.185	0.243	0.387	0.530
Lower Secondary	-0.030	0.385	0.938	-0.287	0.389	0.461
Upper Secondary	-0.527	0.381	0.167			
Post Secondary, Non-Tertiary	-0.368	0.386	0.340			
Bachelor's degree or more	-0.965	0.390	0.013			
Master's degree or PhD	-0.825	0.394	0.036			
Social Class						
Middle	-0.398	0.064	0.000			
High	-0.430	0.229	0.060			
Access	0.477	0.063	0.000			
Female	0.228	0.059	0.000			
Constant		-1.673			-3.885	
Psuedo R^2^		0.209				

n = 5541, Countries: Czech Republic, Estonia, Latvia, Lithuania, Poland, and Slovakia. The proportional odds assumption was violated for Wage Reduction, Staple Consumption Reduction, Luxury Consumption Reduction

Age (55–64, 65+) and Education (Primary, Lower Secondary)

**Table 9:** Partial Proportional Odds Model for Self Rated Health, fourth GNI per capita quartile ($23,910 to $50, 870).

	Good vs. moderate or poor			Good and moderate vs.poor		
	coef.	std. error	p	coef.	std. error	p
**Country Level Variables**						
GNI growth(%)	0.005	0.010	0.660	-0.038	0.017	0.027
**Recession Experience Variables**						
Jobloss-head of household	0.225	0.127	0.076			
Jobloss-other member of household	0.255	0.116	0.028			
Wage Reduction	0.163	0.076	0.033			
Staple Consumption Reduction	0.420	0.091	0.000			
Luxury Consumption Reduction	0.049	0.073	0.504			
**Controls and Socio-Demographics**						
Age						
25–34	0.152	0.183	0.406	0.206	0.167	0.217
35–44	0.206	0.167	0.217			
45–54	0.840	0.161	0.000			
55–64	1.269	0.162	0.000			
65+	1.527	0.165	0.000			
Education						
Primary	0.020	0.172	0.906			
Lower Secondary	-0.359	0.149	0.016			
Upper Secondary	-0.667	0.150	0.000			
Post Secondary, Non-Tertiary	-0.647	0.168	0.000			
Bachelor's degree or more	-0.978	0.172	0.000			
Master's degree or PhD	-1.071	0.194	0.000	-0.386	0.263	0.142
Social Class						
Middle	-0.378	0.082	0.000			
High	-0.666	0.211	0.002			
Access	0.631	0.072	0.000			
Female	0.208	0.071	0.003			
Constant		-1.341			-3.462	
Psuedo R^2^		0.096				

n = 4231, Countries: France, Germany, Great Britain, Italy, Slovenia, and Sweden. The proportional odds assumption was violated for GNIchange, Age (25–34) and Education (Master’s Degree or PhD).

### Pooled Sample

Results for the pooled model for all countries are reported in Table 5. When quartiles are pooled economic growth increases the likelihood of “good” vs. “moderate or poor” self-rated health. Experiencing economic hardship, in general, as the result of the recession is also associated with increased chances of “good” self-rated health with the exception of a loss of luxury consumption. However, wage reduction reduces the likelihood of moderate health.

Among the socio-demographic control variables age increases the likelihood of “good” self-rated health compared to the other categories. For education “no education” is the reference category. The effect of education varies significantly across levels of education and across levels of the dependent variable. Lastly, utilization of healthcare services and being female increase the chances of “good” vs. “moderate or poor” self-rated health, whereas being in the middle and upper social class group is associated with decreased odds of “good” vs. “moderate or poor” self-rated health.

### First GNI per capita quartile

Table 6 provides results for the first disaggregated model, which includes countries that fall in into the first GNI per capita quartile ($1820-$4640). In these nations there is a small, positive effect of growth in GNI per capita which varies across categories of self-rated health (b = 1.0 for “good” vs. “moderate and poor” health and b = .079 for “moderate and good” vs. “poor health”). Loss of staple consumption increases the odds of “good” vs. “moderate or poor” self-rated health while the loss of luxury consumption has the opposite effect. None of the other recession experience variables reach statistical significance. Regarding control variables, age increases respondent’s chances of “good” self-rated health, whereas higher education reduces the likelihood of “good” self-rated health, hence the magnitude of this effect is larger for higher categories of education. The same pattern holds for “good and moderate” vs. “poor” self-rated health. Use of healthcare increases the odds of “good” self-rated health compared to the reference categories (b = .528, p = .000). Respondents of both “middle” and “high” self-assessed social class are less likely to report “good” self-rated health than those with “low” self-assessed social class.

### Second GNI per capita quartile

Results for the portion of the sample that fall in the second GNI per capita quartile ($5550-$10,100) are shown in Table 7. Here, the effect of economic growth is highly asymmetrical across categories of self-rated health; the coefficients for GNIchange indicate that economic growth increases the likelihood of “good” self-rated health while it has negative effect on the odds of “good or moderate” vs. “poor” health. In addition, the effect on “good” self-rated health is statistically significant, but the effect on “good or moderate” self-rated health is not at conventional alpha levels (e.g. p = .05).

Among the recession experience variables wage reduction is the only variable does not reach statistical significance (p = .910). Each of the other variables, with the exception of Luxury Consumption Reduction, is positively associated with the likelihood of reporting “good” self-rated health relative to the other categories. For the control variables age has a differential effect across categories but, in general, the direction of this effect is the same for both “good” and “good and moderate” self-rated health. Education and self-assessed social class at all levels is negatively associated with the chances of “good” self-rated health, while household use of medical care and being female have a positive effect on “good” self-rated health relative to the other categories of self-rated health.

### Third GNI per capita quartile

Table 8 provides the results of the partial proportional odds model for the portion of the sample falling in the third GNI per capita quartile ($12,330-$18,450). For residents of these countries economic growth decreases the likelihood of reporting “good” self-rated health (b = -.116) while the effect on “good or moderate” self-rated health is positive but not statistically significant (p = .351). Regarding the recession experience variables we find a similar pattern as that demonstrate in our previous models: most recession experiences increase the likelihood that a respondent will report “good” self-rated health though a job loss for another member of the household and a wage cut do not approach statistical significance. The lone exception again is a loss of luxury consumption, which has a negative effect on the likelihood of “good” self-rated health and is approaching significance (p = .075). Unlike previous models, the proportional odds assumption was violated for two of the recession experience variables. A loss of wages has a statistically significant and negative effect on the odds of reporting “good or moderate” health (b = -.303) while a loss of staple consumption has a positive effect on the odds of this category (b = .470).

Among the control variables, Age shows a similar effect as in previous models. Generally, older age is associated with increased chances of “good” self-rated health. The opposite holds true of education: as education increases the likelihood of “good” self-rated health decreases. We also find that self-assessed social class reduces the chances of “good” self-rated health while household use of medical care and being female as associated with increased odds of reporting “good” self-rated health.

### Fourth GNI per capita quartile

Results for the countries in the fourth GNI per capita quartile ($23,910 to $50,870) are reported in Table 9. As with the prior model the effect of economic growth is asymmetrical. In these countries GNI per capita growth has no association with the odds of “good” self-rated health and a negative effect on “moderate” self-rated health.

Compared to prior models the recession experiences have a more consistent effect. The coefficient for each is positive, and none violate the proportional odds assumption. Unlike previous models, the effect of a luxury consumption loss is not statistically significant (p = .504). Among the control variables, increases in age increase the likelihood of “good” vs. “moderate ad poor” self-rated health. Only a few of the education categories reach statistical significance. Subjective class has a negative relationship with “good” self-rated health while the effect of use of healthcare and being female are positive. Compared to other models this model has achieved the lowest pseudo R-squared (.096).

### Predicted Probabilities

In this section we report predicted probabilities derived from our partial proportional odds models. The coefficients reported in Tables 4–8 describe the effect of each variable on the chances of “good” or “moderate and good” relative to other categories of the dependent variable; it is possible that a variable might have a positive effect relative to a set of references groups but not to another set. However, simultaneously interpreting dozens of regression coefficients for multiple thresholds of self-rated health simultaneously is challenging. Predicted probabilities provide a simple way of better understanding the complex modelling results.

First, we calculated a predicted probability for each case at its observed values of all independent variables using the coefficients reported in Tables 4–8 for each strata of countries. Then, we created two archetypal groups of respondents: those experiencing the worst effects of recession and those reporting no negative experience. The “worst effects” archetype experienced a job loss for the head of the household, a personal job loss, a wage reduction, and a reduction in the consumption of both staple and luxury goods. The “no effects” archetype had none of these experiences. For both of these archetypes all other covariates (e.g. education, age, sex) are held at their observed values. Table 10 reports averaged probabilities for the “no effects” and “worst effects” archetypal groups for each GNI per capita quartile.

**Table 10:** Predicted probabilities of Self-Rated health by recession experiences.

		Good	Moderate	Poor
All Countries				
	worst effects	0.425	0.408	0.167
	No effects	0.594	0.299	0.299
First GNI per capita quartile ($1820-$4640)				
	worst effects	0.518	0.336	0.146
	No effects	0.592	0.300	0.109
Second GNI per capita quartile ($5550-$10,100)				
	worst effects	0.431	0.394	0.175
	No effects	0.543	0.342	0.115
Third GNI per capita quartile ($12,330-$18,450)				
	worst effects	0.385	0.436	0.179
	No effects	0.485	0.381	0.134
Fourth GNI per capita quartile ($23,910-$50,870)				
	worst effects	0.492	0.351	0.157
	No effects	0.717	0.222	0.061

The predicted probabilities calculated from the pooled model indicate that the cumulative effect of recession experiences is to decrease the probability of “good” or “poor” self-rated health while increasing the probability of “moderate” self-rated health. Turning to the lowest income nations (i.e. the first GNI per capita nations) we note that the difference in probability between the two archetypes is substantively small across all three categories of the dependent variable. For the countries in the second GNI per capita stratum individuals who experienced no ill effects of the recession are more likely to report “good” self-rated health and less likely to report “moderate” and “poor” health though the difference is small. Among the countries in the third GNI per capita quartile negative recession experiences reduce the chances of “good” self-rated health while increasing the chances of “poor” or “moderate” health compared to those with “no effects”. In these countries the difference in predicted probabilities between the two archetypes is larger than in the first two groups of countries. Turning to the highest income nations, those who have experienced no effects of the recession are much more likely to report “good” self-rated and far less likely to report “moderate” or “poor” health. Indeed, the average probability of “poor” self-rated health is very small (.061). Notably, in each GNI per capita strata the probability of “good” self-rated health is higher than the other categories.

Very few respondents endured all of the recession experiences. To better understand the unique effect of each recession experience we report averaged predicted probabilities for these variables organized by GNI per capital quartiles in Table 11; in the interest of parsimony we only report the probability of “Good” self-rated health. These probabilities are calculated for each case at their observed values of all predictor variables. For the pooled model most of the recession experiences have a negligible effect. The loss of staple consumption has a substantively important downward effect on the average probability of “Good” self-rated health while the loss of luxury consumption has a small positive effect. This pattern roughly holds across all of the GNI per capita groups; job losses and wage reduction have small or null effects while the effect of a staple consumption loss is substantively large in each group. Overall, the effect of the recession experiences variables is largest among the high-income nations and smallest in the low-income nations.

**Table 11:** Predicted Probability of "Good" Self-Rated Health by Specific Recession Experiences.

	All countries	First GNI per capita quartile ($1820-$4640)	Second GNI per capita quartile ($5,550-$10,100)	Third GNI per capita quartile ($12,330-$18,450)	Fourth GNI per capita quartile ($23,910-$50,870)
Job Loss-Head of Household					
No	0.55	0.61	0.51	0.52	0.64
Yes	0.51	0.61	0.48	0.52	0.59
Job Loss-Other member of household					
No	0.55	0.62	0.52	0.52	0.65
Yes	0.51	0.59	0.48	0.51	0.58
Wage Reduction					
No	0.54	0.60	0.49	0.49	0.63
Yes	0.55	0.63	0.54	0.56	0.64
Staple Consumption Reduction					
No	0.62	0.67	0.58	0.57	0.70
Yes	0.43	0.52	0.40	0.44	0.53
Luxury Consumption Reduction					
No	0.52	0.58	0.48	0.48	0.63
Yes	0.57	0.64	0.54	0.55	0.64

## Discussion

This paper demonstrates that recessions and macroeconomic growth have a very complex effect on self-rated health. In the pooled sample and for the countries in the lowest GNI per capital quartile macroeconomic growth increases the odds of both “good” and “good and moderate” health while for countries in the second and third GNI per capital quartiles its effect varies substantially across categories of self-rated health. For high income nations (e.g. fourth GNI per capita quartile) economic growth does not increase the odds of “good” self-rated health and reduces the odds of “moderate” health. Overall, these results are consistent with prior research that demonstrates that economic growth in developing countries improves health, but has the opposite effect in developed countries [9]. These findings on the differential effects of macro-economic growth are consistent with the argument that stronger social safety nets in developed countries attenuate the effect of macroeconomic recession on health [25]. Our findings are somewhat more nuanced as economic growth increases the chances of both “good” and “good and moderate” self-rated health among the lowest income nations while it has no effect on the odds of “good” self-rated health and *reduces* the odds of “good and moderate” self-rated health among high-income nations. Overall, it appears that the effects of macroeconomic conditions on self-rated health are highly non-linear. The partial proportional odds modelling strategy pursued in this paper allows us to detect these nuances, suggesting that this approach may be useful in future research on the relationship between economic conditions and health.

Among the individual-level recession experience variables we find very mixed results. For the lower income countries (i.e. Table 5 and Table 6) recession experience variables are not all statistically significant and have substantively small effects; these effects where further corroborated by the predicted probabilities reported in Tables 10 and 11. Hence, for these nations a loss of a job or a loss of wages may not be a strong predictor of self-rated health. The lone exceptions are changes in consumption. Indeed, a loss of staple consumption retains statistical significance across all model specifications and, we shown in Table 10, has a stronger effect than the other recession experiences. The magnitude of the coefficient varies substantially across GNI per capita quartiles, which suggests that national-level characteristics may to some extent modify how individual-level effects of recessions determine self-rated health That is, individual or household economic experiences appear to be important predictors of health in high income nations, but are relatively unimportant in low-income nations. Taken together with our country-level findings discussed in the previous paragraph it appears that country-level macroeconomic conditions are most relevant for health in lower income nations while individual or household economic circumstances are most relevant for health in higher income nations. Still, we note that the effect of individual and household economic experiences is not especially large. To some degree these results are not consistent with prior research which has shown a significant downward health effect of negative economic experiences [10, 16,17]; this difference between the established literature and this paper could be because many of the prior studies examined only a single country.

The socio-demographic variables are conceptualized as controls in this analysis. Still, their effects merit further discussion as they may not be consistent with conventional wisdom. For instance, we find that education typically lowers the probability of “good” versus “moderate or poor” health. We also find that educational predictors often violate the proportional odds assumption, indicating that its effects vary across categories of self-rated health; these findings may be counter to other studies. There are a few major reasons why the results reported here may not be entirely consistent with conventional wisdom regarding the effects of socio-demographic predictors. First, the partial proportional odds model can unveil more complex relationships than more simplistic, yet more common, modelling strategies like dichotomizing self-rated health and estimating a binary logistic regression as this strategy involves a significant loss of information. Secondly, our socio-demographic predictors are all categorical and it is relatively common for scholars to treat categorical predictors as continuous, which can potentially mask complex, non-linear effects. Thirdly, many of the countries included in this analysis have received relatively little study in the literature and it may be not possible to generalize findings about the effects of socio-demographic predictors from other countries to these nations.

As with all research there are several limitations to this study. Perhaps the most significant limitation concerns issues of time. The recession began in late 2007 in the U.S. and effected different countries at different times and some countries never formally entered recession (i.e. two consecutive quarters of no or negative economic growth). Our recession experience variables are constructed from questions which ask respondents about their household’s experiences, but they do not assess how long ago the particular experience occurred or for how long it had endured. For example, a temporary job loss is unlikely to have the same deleterious effect on health as an ongoing, long-term job loss. Future studies could improve upon this research by considering both economic experiences and the duration of those experiences. A further limitation is the response rate, which varied significantly by country. While our results are adjusted via sampling weights there could be unknown problems of non-response bias lurking within the data. A third limitation relates to potential validity problems with self-rated health when studied cross-nationally. Understandings of health are formed in culturally-specific contexts and, for this reason, some groups may interpret questions about health differently than others [45,46]. This could also undetectable problems of validity as respondents in different countries, or of different cultural groups, might not interpret questions about self-rated health in the same way. A fourth limitation relates to missing data. A small group of respondents (161 cases or 0.8% of the pooled sample) indicated that they “don’t know” their health status and these respondents were excluded from the analysis-it is possible that the exclusion of these cases may have produced a small amount of bias in the results reported above. In an unreported analysis, we compared the “don’t know” responses to the valid cases on the socio-demographic predictors (i.e. age, education, gender and subjective social class) and found no substantive differences between these two groups. For instance, the modal education for the “don’t know” group was Upper Secondary- the mode the valid cases is the adjacent category- Post-Secondary

## Conclusion

Self-rated health is determined by a number of economic factors ranging from personal or household-level experience to macroeconomic growth to GNI per capita. However, the effect of both micro- and macro-level variables varies substantially across countries of different levels of economic development. In particular, national economic conditions appear most important in low-income nations while personal and household economic conditions are more important in high-income nations. In addition, the effect of these variables is not always consistent across categories of self-rated health. Future research, possibly using proportional odds models and longitudinal data, is needed to better understand these relationships.

## Supporting Information

S1 Appendix Percentage distribution of all the control variables by country. (DOCX)

S2 Appendix Wald tests for the proportional odds assumption. A significant test (e.g. p < .05) indicates that the proportional odds assumption has been violated.
(DOCX)

S3 Appendix Multicollinearity Diagnostics. (DOCX)
