# Supplementary material for: Understanding Recession and Self-Rated Health with the Partial Proportional Odds Model: An Analysis of 26 Countries
Source: PLoS One. 2015 Oct 29;10(10):e0140724. doi: 10.1371/journal.pone.0140724 (PMC4626113; doi:10.1371/journal.pone.0140724)
Supplement: S1 Appendix — (DOCX) [file pone.0140724.s001.docx]

|  | **Age** | | | | | |  |
| --- | --- | --- | --- | --- | --- | --- | --- |
|  | 18-24 | 25-34 | 35-44 | 45-54 | 55-64 | 65+ |  |
|  | % | % | % | % | % | % |  |
| Albania | 17 | 18 | 20 | 23 | 12 | 10 |  |
| Belarus | 19 | 27 | 20 | 15 | 12 | 7 |  |
| Bosnia | 13 | 24 | 17 | 17 | 15 | 15 |  |
| Bulgaria | 6 | 14 | 17 | 19 | 18 | 26 |  |
| Croatia | 7 | 17 | 17 | 16 | 19 | 24 |  |
| Czech Republic | 8 | 21 | 19 | 20 | 17 | 13 |  |
| Estonia | 12 | 14 | 12 | 12 | 17 | 32 |  |
| France | 5 | 12 | 20 | 20 | 21 | 22 |  |
| Germany | 6 | 15 | 20 | 23 | 17 | 19 |  |
| Great Britain | 8 | 14 | 17 | 16 | 17 | 28 |  |
| Hungary | 7 | 13 | 15 | 15 | 20 | 30 |  |
| Italy | 8 | 14 | 25 | 21 | 16 | 16 |  |
| Kosovo | 25 | 29 | 20 | 11 | 9 | 6 |  |
| Latvia | 10 | 15 | 14 | 18 | 14 | 29 |  |
| Lithuania | 9 | 10 | 16 | 19 | 17 | 29 |  |
| Macedonia | 13 | 21 | 20 | 19 | 13 | 15 |  |
| Moldova | 9 | 14 | 15 | 19 | 20 | 23 |  |
| Montenegro | 16 | 26 | 20 | 15 | 11 | 11 |  |
| Poland | 8 | 20 | 17 | 16 | 20 | 20 |  |
| Romania | 8 | 16 | 17 | 16 | 17 | 26 |  |
| Russia | 11 | 20 | 16 | 15 | 20 | 18 |  |
| Serbia | 8 | 16 | 16 | 19 | 21 | 21 |  |
| Slovakia | 10 | 25 | 24 | 23 | 12 | 6 |  |
| Slovenia | 13 | 20 | 16 | 20 | 15 | 16 |  |
| Sweden | 3 | 11 | 22 | 19 | 24 | 21 |  |
| Ukraine | 11 | 20 | 17 | 15 | 16 | 21 |  |
|  |  |  |  |  |  |  |  |
|  | **Education** | | | | | | |
|  | No education | Primary | Lower Secondary | Upper Secondary | Post Secondary, Non-Tertiary | Bachelor's degree or more | Master's degree or PhD |
|  | % | % | % | % | % | % | % |
| Albania | 3 | 21 | 15 | 39 | 5 | 17 | 1 |
| Belarus | 1 | 1 | 4 | 14 | 42 | 8 | 30 |
| Bosnia | 8 | 18 | 23 | 37 | 5 | 9 | 1 |
| Bulgaria | 1 | 4 | 17 | 23 | 34 | 14 | 9 |
| Croatia | 7 | 9 | 10 | 52 | 2 | 20 | 1 |
| Czech Republic | 0 | 7 | 34 | 42 | 4 | 4 | 9 |
| Estonia | 0 | 16 | 6 | 31 | 25 | 12 | 9 |
| France | 15 | 9 | 14 | 12 | 17 | 19 | 14 |
| Germany | 2 | 2 | 32 | 40 | 12 | 8 | 5 |
| Great Britain | 8 | 2 | 29 | 24 | 19 | 15 | 4 |
| Hungary | 1 | 27 | 27 | 17 | 14 | 10 | 5 |
| Italy | 1 | 12 | 29 | 45 | 1 | 3 | 9 |
| Kosovo | 16 | 15 | 11 | 36 | 17 | 5 | 0 |
| Latvia | 0 | 11 | 8 | 37 | 23 | 17 | 4 |
| Lithuania | 1 | 23 | 24 | 25 | 8 | 14 | 5 |
| Macedonia | 6 | 27 | 8 | 31 | 12 | 15 | 1 |
| Moldova | 1 | 8 | 52 | 3 | 18 | 18 | 0 |
| Montenegro | 3 | 11 | 12 | 51 | 9 | 13 | 1 |
| Poland | 1 | 15 | 24 | 28 | 15 | 9 | 8 |
| Romania | 7 | 22 | 20 | 24 | 8 | 15 | 3 |
| Russia | 1 | 2 | 8 | 18 | 41 | 29 | 1 |
| Serbia | 4 | 16 | 18 | 41 | 9 | 10 | 1 |
| Slovakia | 3 | 5 | 25 | 45 | 3 | 4 | 15 |
| Slovenia | 2 | 15 | 18 | 43 | 8 | 13 | 1 |
| Sweden | 5 | 2 | 10 | 10 | 15 | 25 | 34 |
| Ukraine | 1 | 3 | 8 | 15 | 39 | 33 | 1 |
|  |  |  |  |  |  |  |  |
|  | **Social Class** | | |  |  |  |  |
|  | Low | Middle | High |  |  |  |  |
|  | % | % | % |  |  |  |  |
| Albania | 19 | 69 | 12 |  |  |  |  |
| Belarus | 24 | 74 | 2 |  |  |  |  |
| Bosnia | 24 | 72 | 4 |  |  |  |  |
| Bulgaria | 44 | 54 | 3 |  |  |  |  |
| Croatia | 27 | 67 | 6 |  |  |  |  |
| Czech Republic | 15 | 81 | 4 |  |  |  |  |
| Estonia | 28 | 71 | 2 |  |  |  |  |
| France | 23 | 73 | 4 |  |  |  |  |
| Germany | 19 | 75 | 5 |  |  |  |  |
| Great Britain | 30 | 65 | 5 |  |  |  |  |
| Hungary | 42 | 57 | 1 |  |  |  |  |
| Italy | 14 | 80 | 6 |  |  |  |  |
| Kosovo | 19 | 74 | 7 |  |  |  |  |
| Latvia | 47 | 52 | 1 |  |  |  |  |
| Lithuania | 38 | 60 | 2 |  |  |  |  |
| Macedonia | 37 | 61 | 2 |  |  |  |  |
| Moldova | 34 | 62 | 4 |  |  |  |  |
| Montenegro | 16 | 79 | 4 |  |  |  |  |
| Poland | 19 | 73 | 8 |  |  |  |  |
| Romania | 41 | 57 | 1 |  |  |  |  |
| Russia | 42 | 56 | 2 |  |  |  |  |
| Serbia | 42 | 56 | 2 |  |  |  |  |
| Slovakia | 12 | 83 | 6 |  |  |  |  |
| Slovenia | 26 | 71 | 3 |  |  |  |  |
| Sweden | 11 | 80 | 9 |  |  |  |  |
| Ukraine | 49 | 50 | 0 |  |  |  |  |
|  |  |  |  |  |  |  |  |
|  | **Access** | |  |  |  |  |  |
|  | No | Yes |  |  |  |  |  |
|  | % | % |  |  |  |  |  |
| Albania | 38 | 62 |  |  |  |  |  |
| Belarus | 21 | 79 |  |  |  |  |  |
| Bosnia | 40 | 60 |  |  |  |  |  |
| Bulgaria | 23 | 77 |  |  |  |  |  |
| Croatia | 27 | 73 |  |  |  |  |  |
| Czech Republic | 29 | 71 |  |  |  |  |  |
| Estonia | 38 | 62 |  |  |  |  |  |
| France | 62 | 38 |  |  |  |  |  |
| Germany | 56 | 44 |  |  |  |  |  |
| Great Britain | 37 | 63 |  |  |  |  |  |
| Hungary | 48 | 52 |  |  |  |  |  |
| Italy | 48 | 52 |  |  |  |  |  |
| Kosovo | 22 | 78 |  |  |  |  |  |
| Latvia | 18 | 82 |  |  |  |  |  |
| Lithuania | 24 | 76 |  |  |  |  |  |
| Macedonia | 17 | 83 |  |  |  |  |  |
| Moldova | 17 | 83 |  |  |  |  |  |
| Montenegro | 38 | 62 |  |  |  |  |  |
| Poland | 43 | 57 |  |  |  |  |  |
| Romania | 39 | 61 |  |  |  |  |  |
| Russia | 22 | 78 |  |  |  |  |  |
| Serbia | 30 | 70 |  |  |  |  |  |
| Slovakia | 27 | 73 |  |  |  |  |  |
| Slovenia | 26 | 74 |  |  |  |  |  |
| Sweden | 35 | 65 |  |  |  |  |  |
| Ukraine | 30 | 70 |  |  |  |  |  |
|  |  |  |  |  |  |  |  |
|  | **Female** | |  |  |  |  |  |
|  | Male | Female |  |  |  |  |  |
|  | % | % |  |  |  |  |  |
| Albania | 45 | 55 |  |  |  |  |  |
| Belarus | 38 | 62 |  |  |  |  |  |
| Bosnia | 43 | 57 |  |  |  |  |  |
| Bulgaria | 37 | 63 |  |  |  |  |  |
| Croatia | 44 | 56 |  |  |  |  |  |
| Czech Republic | 39 | 61 |  |  |  |  |  |
| Estonia | 29 | 71 |  |  |  |  |  |
| France | 48 | 52 |  |  |  |  |  |
| Germany | 43 | 57 |  |  |  |  |  |
| Great Britain | 44 | 56 |  |  |  |  |  |
| Hungary | 40 | 60 |  |  |  |  |  |
| Italy | 34 | 66 |  |  |  |  |  |
| Kosovo | 43 | 57 |  |  |  |  |  |
| Latvia | 41 | 59 |  |  |  |  |  |
| Lithuania | 33 | 67 |  |  |  |  |  |
| Macedonia | 45 | 55 |  |  |  |  |  |
| Moldova | 36 | 64 |  |  |  |  |  |
| Montenegro | 45 | 55 |  |  |  |  |  |
| Poland | 47 | 53 |  |  |  |  |  |
| Romania | 43 | 57 |  |  |  |  |  |
| Russia | 30 | 70 |  |  |  |  |  |
| Serbia | 44 | 56 |  |  |  |  |  |
| Slovakia | 38 | 62 |  |  |  |  |  |
| Slovenia | 44 | 56 |  |  |  |  |  |
| Sweden | 54 | 46 |  |  |  |  |  |
| Ukraine | 30 | 70 |  |  |  |  |  |
