# Supplementary material for: Understanding Recession and Self-Rated Health with the Partial Proportional Odds Model: An Analysis of 26 Countries
Source: PLoS One. 2015 Oct 29;10(10):e0140724. doi: 10.1371/journal.pone.0140724 (PMC4626113; doi:10.1371/journal.pone.0140724)
Supplement: S2 Appendix — A significant test (e.g. p < .05) indicates that the proportional odds assumption has been violated. (DOCX) [file pone.0140724.s002.docx]

|  | Model 1 | Model 2 | Model 3 | Model 4 | Model 5 |
| --- | --- | --- | --- | --- | --- |
| **Country Level Variables** |  |  |  |  |  |
| GNIchange | 0.000 | 0.025 | 0.000 | 0.000 | 0.008 |
|  |  |  |  |  |  |
| **Recession Experience Variables** | |  |  |  |  |
| Jobloss-head of household | 0.729 | 0.748 | 0.823 | 0.819 | 0.526 |
| Jobloss-other member of household | 0.480 | 0.886 | 0.889 | 0.901 | 0.985 |
| Wage Reduction | 0.000 | 0.444 | 0.119 | 0.000 | 0.145 |
| Staple Consumption Reduction | 0.374 | 0.430 | 0.899 | 0.042 | 0.117 |
| Luxury Consumption Reduction | 0.413 | 0.698 | 0.253 | 0.383 | 0.878 |
|  |  |  |  |  |  |
| **Controls and Socio-Demographics** | |  |  |  |  |
| Age |  |  |  |  |  |
| 25-34 | 0.780 | 0.254 | 0.715 | 0.367 | 0.834 |
| 35-44 | 0.264 | 0.754 | 0.024 | 0.566 | 0.874 |
| 45-54 | 0.915 | 0.002 | 0.009 | 0.642 | 0.720 |
| 55-64 | 0.285 | 0.781 | 0.016 | 0.012 | 0.665 |
| 65+ | 0.107 | 0.738 | 0.651 | 0.001 | 0.160 |
|  |  |  |  |  |  |
| Education |  |  |  |  |  |
| Primary | 0.001 | 0.302 | 0.426 | 0.040 | 0.152 |
| Lower Secondary | 0.000 | 0.671 | 0.147 | 0.028 | 0.184 |
| Upper Secondary | 0.000 | 0.040 | 0.226 | 0.266 | 0.096 |
| Post Secondary, Non-Tertiary | 0.000 | 0.000 | 0.007 | 0.121 | 0.177 |
| Bachelor's degree or more | 0.000 | 0.000 | 0.149 | 0.139 | 0.619 |
| Master's degree or PhD | 0.000 | 0.037 | 0.098 | 0.206 | 0.001 |
|  |  |  |  |  |  |
| Social Class |  |  |  |  |  |
| Middle | 0.002 | 0.615 | 0.018 | 0.931 | 0.608 |
| High | 0.734 | 0.853 | 0.068 | 0.554 | 0.176 |
|  |  |  |  |  |  |
| Access | 0.166 | 0.832 | 0.809 | 0.170 | 0.177 |
|  |  |  |  |  |  |
| Female | 0.032 | 0.019 | 0.012 | 0.231 | 0.176 |
