# Supplementary material for: Understanding Recession and Self-Rated Health with the Partial Proportional Odds Model: An Analysis of 26 Countries
Source: PLoS One. 2015 Oct 29;10(10):e0140724. doi: 10.1371/journal.pone.0140724 (PMC4626113; doi:10.1371/journal.pone.0140724)
Supplement: S3 Appendix — (DOCX) [file pone.0140724.s003.docx]

|  | Variance Inflation Factor | Tolerance |
| --- | --- | --- |
| GNIchange2010 | 1.04 | 0.96 |
| Jobloss-head of household | 1.03 | 0.95 |
| Jobloss-other member of household | 1.05 | 0.97 |
| Wage Reduction | 1.05 | 0.95 |
| Staple Consumption Reduction | 1.11 | 0.88 |
| Luxury Consumption Reduction | 1.04 | 0.96 |
| Age | 1.10 | 0.91 |
| Education | 1.08 | 0.93 |
| Social Class | 1.12 | 0.89 |
| Female | 1.01 | 0.99 |
| Access | 1.02 | 0.98 |
